# Overpressure Exposure From .50-Caliber Rifle Training Is Associated With Increased Amyloid Beta Peptides in Serum

**DOI:** 10.3389/fneur.2020.00620

**Published:** 2020-07-24

**Authors:** Bharani Thangavelu, Christina R. LaValle, Michael J. Egnoto, Jeffrey Nemes, Angela M. Boutté, Gary H. Kamimori

**Affiliations:** ^1^Brain Trauma Neuroprotection Branch, Center for Military Psychiatry and Neuroscience, Walter Reed Army Institute of Research, Silver Spring, MD, United States; ^2^Blast Induced Neurotrauma Branch, Center for Military Psychiatry and Neuroscience, Walter Reed Army Institute of Research, Silver Spring, MD, United States

**Keywords:** .50 caliber rifle training, overpressure, low-level blast, occupational exposure, traumatic brain injury, serum biomarkers, amyloid beta peptides

## Abstract

**Background:** Overpressure (OP) is an increase in air pressure above normal atmospheric levels. Military personnel are repeatedly exposed to low levels of OP caused by various weapon systems. Repeated OP may increase risk of neurological disease or psychological disorder diagnoses. A means to detect early phase effects that may be relevant to brain trauma remain elusive. Therefore, development of quantitative and objective OP-mediated effects during acute timeframes would vastly augment point-of-care or field-based decisions. This pilot study evaluated the amplitude of traumatic brain injury (TBI)–associated biomarkers in serum as a consequence of repeated OP exposure from .50-caliber rifle use over training multiple days.

**Objective:** To determine the acute temporal profile of TBI-associated serum biomarkers and their relationship with neurocognitive decrements or self-reported symptoms among participants exposed to low-level, repeated OP from weapons used in a training environment.

**Methods:** Study participants were enrolled in .50-caliber sniper rifle training and exposed to mild OP (peak pressure 3.8–4.5 psi, impulse 19.27–42.22 psi-ms per day) for three consecutive days (D1–D3). Defense automated neurobehavioral assessment (DANA) neurocognitive testing, symptom reporting, and blood collection were conducted 2–3 h before (pre-) and again 0.45–3 h after (post-) OP exposure. The TBI-associated serum biomarkers, glial fibrillary acidic protein (GFAP), ubiquitin C-terminal hydrolase-L1 (UCH-L1), neurofilament light (Nf-L), tau, and amyloid beta peptides (Aβ-40 and Aβ-42) were measured using digital ELISAs.

**Results:** Serum GFAP decreased on D1 and D3 but not D2 after OP exposure. Nf-L was suppressed on D3 alone. Aβ-40 was elevated on D2 alone while Aβ-42 was elevated each day after OP exposure. Suppression of GFAP and elevation of Aβ-42 correlated to OP-mediated impulse levels measured on D3.

**Conclusions:** Acute measurement of Aβ-peptides may have utility as biomarkers of subconcussive OP caused by rifle fire. Fluctuation of GFAP, Nf-L, and particularly Aβ peptide levels may have utility as acute, systemic responders of subconcussive OP exposure caused by rifle fire even in the absence of extreme operational deficits or clinically defined concussion.

## Introduction

Overpressure (OP) is an increase in air pressure above atmospheric levels (1, 2). Military and law enforcement personnel are routinely exposed to OP within training environments and in-theater. OP-mediated effects are most widely understood in the context of moderate-to-high levels of blast caused by improvised explosive devices (IEDs) (3–5). However, operational training involving .50-caliber rifle systems are also capable of generating repeated OP exposure (6). OP levels consistently exceed 4 psi, an “above safe” level, which is dependent upon rifle configuration (e.g., muzzle devices and ammunition type) as well as environment. For e.g., prone firing positions and shots taken from atop hard surfaces (e.g., vehicles or concrete) are associated with even greater OP levels (7).

OP exposure can cause brain trauma and may start underlying pathology that leads to neurodegenerative disorders (8); however, the exposure thresholds at which injuries occur are not yet defined. Blast-related mild traumatic brain injury (mTBI) is often identified by symptoms, but there is concern that blast exposures that do not result in symptom reporting or mTBI, referred to as subconcussive exposures, may also be associated with neurotrauma (9). Effects of repeated OP exposure have been measured and include neurocognitive decrements, blood-based biomarker level changes, and manifestation of symptoms (10), which are similar to those observed among mild traumatic brain (mTBI) or concussed patients. These effects are observed in absence of a diagnosable injury, and personnel remain fit for duty. Symptom reporting among operators with repetitive blast exposures are characterized, in part, by headaches, dizziness, taking longer to think, and tinnitus (11–13). Detection, prevention, or mitigation of these subconcussive, subacute, and chronic outcomes may be met by assessment of OP-mediated health effects that are measured acutely. Symptomology is often variable, subjective, and may be underreported (14, 15). Therefore, objective measurements that identify OP-mediated health effects present an unmet operational need and have become a health care priority (https://www.congress.gov/bill/115th-congress/senate-bill/2883/all-info) such that routine monitoring of health-related effects from operational exposure has become a topic of interest.

The use of blood-based biomarkers may augment the ability to objectively measure the effects of OP inclusive of neurocognitive decrement and concussion-like symptoms. Several proteins of the central nervous system (CNS) are used to identify TBI and neurological disease. Glial fibrillary acidic protein (GFAP) is an abundant astroglial protein within the cytoskeleton (16). Ubiquitin C-terminal hydrolase-L1 (UCH-L1) is a deubiquitinating enzyme enriched in neuronal cell bodies (17). GFAP and UCH-L1 are the most widely used biomarkers for acute, moderate-to-severe TBI (18–21). Tau, an important microtubule-associated structural element of the neuronal cytoskeleton (22). Neurofilament light chain (Nf-L), another central nervous system–enriched protein, is also a component of the axonal cytoskeleton that is primarily expressed in large-caliber, myelinated axons. Nf-L has been identified in peripheral blood collected weeks or months after TBI (23). Tau and Nf-L are currently the most widely used biomarkers applied to acute–subacute mTBI or concussion with variable results (24–27). Amyloid precursor protein (APP), an integral membrane protein predominantly expressed in the synapses of neurons, is the precursor of amyloid beta (Aβ) peptides, which have been shown to be elevated in blood of both patients and animal models after TBI (28). Overall, evaluation of serum-based biomarkers are capable of providing objective measures.

We sought to determine if acute assessment of these TBI-associated proteins were applicable to low levels of OP caused by weapons use. This study measured serum biomarker levels (GFAP, tau, Nf-L, UCH-L1, and Aβ peptides), reaction time (a metric of neurocognitive performance), and concussion-like symptomology among military personnel exposed to mild, repeated OP caused by .50-caliber rifle discharge. Early and objective quantitation of OP-mediated peripheral biomarkers may aid in rapid decision making and identify systemic effects even in the absence of a definitive mTBI or concussion diagnosis.

## Methods

### Study Participants

Male, active-duty, law-enforcement personnel (*n* = 15) engaged in a 3-day (D)-long training course within a single site. The daily interval of training was ~24 h, during which .50-caliber rifle firing, neurocognitive testing, blood sampling, and symptomology assessment were conducted before and after each daily training session. Informed consent was obtained prior to all procedures and testing. The protocol was approved by the Walter Reed Army Institute of Research Institutional Review Board (WRAIR protocol #2304). All participants were assigned unique identification numbers such that data was deidentified prior to collecting field-based metrics, symptom surveys, and biological samples used in this study. All outcome metrics, including details regarding rifle systems, are indicated for each study participant (Supplementary Table 1). Rifle systems, configurations, and ammunition types varied slightly between participants based on departmental resources, but all systems ranged from 20- to 29-inch barrels, generally fired unsuppressed, using a mixture of 690–750 grain (gr) ammunition. The most common ammunition was Hornady .50 caliber AMAX 750 gr, and the most common rifle systems were the Barrett M82A1 20- and 29-inch configuration or the M107A1. Most systems were semiautomatic and can have a different pressure signature at the ear due to the cycling of the action than other systems with some bolt-action platforms, and no uniform trends were noted, thus prohibiting grouped analyses based on barrel length. At pre- and post-OP exposure time-points, participants completed DANA tasks, blood collection, and symptom survey assessment. Participants did not report adverse health conditions, e.g., dehydration, that would be cause for removal from the study. Weather conditions were moderate and did not affect the study or the participants.

### Overpressure Measurements

OP measurements were conducted as previously described (29). All participants were exposed to 3-days of OP events, firing 4–50 rounds per day from a variety of positions (prone, seated, kneeling, standing, and supported standing from barricade). All training days were consecutive. OP was measured as pounds per square inch (psi) using the B3 Blast Gauge sensor (generation 6, BlackBox Biometrics, Rochester, NY) mounted on the left shoulder of each participant. The sensor from the left shoulder best approximates incident orientation to the blast wave (30). The B3 is a small, lightweight, accurate, disposable, and off-the-shelf exposure measurement device that can assess OP and impulse exposures for a blast event; the sensors are positioned on the subject such that they best approximate incident orientation. It has been developed to be worn by participants in complex environments and assess OP exposure. It records and collects data from the blast wave: overpressure, acceleration (rate at which speed changes), and impulse (time exposed to certain levels of OP). Participants are static during training; the B3 sensor are not designed to measure motion or movement of the user (e.g., study participant). Peak pressure is the maximum overpressure peak recorded by the B3 during a blast event per individual per day. Cumulative impulse (psi-ms) is derived from the summation of psi-ms signals per participant per day. Overpressure (psi) and impulse (psi multiplied by milliseconds [psi-ms]) are displayed for each incident and used to calculate the peak overpressure and cumulative impulse values for the training session (Supplementary Table 1).

### Assessment of Neurocognitive Performance

The defense automatized neurocognitive assessment (DANA) tool was administered prior to (pre-OP: −3 to −2 h) and after (post-OP: +0.45 to +3 h) OP exposure to mirror blood-draw time. The DANA consists of three subtasks conducted with a hand-held device and monitor screen. (1) Simple reaction time (SRT) measures pure reaction time when the participant was required to tap on the location of the yellow asterisk symbol as quickly as possible each time it appeared. (2) Procedural reaction time (PRT) is a choice reaction time that measures accuracy, reaction time, and impulsivity. The screen displays one of four numbers for 3 s. The participant is required to press on a left button (“2” or “3”) or right button (“4” or “5”) depending on the number pressed. This choice reaction time task targets simple executive functioning with easy decision-making capabilities. (3) Go-no-go (GNG) is a forced choice reaction-time task. A picture of a house is presented on the screen. Either a “friend” (green) or “foe” (white) appear in a window. The respondent must push a “fire” button only when a “foe” appears. The choice reaction time measures sustained attention and impulsivity. The test quantifies speed and accuracy of target omissions and commissions.

### Symptom Reporting

Participants completed a 32-item, paper-and-pencil health symptom survey before (pre-) and after (post-) OP exposure within the same timeframe as DANA assessment and blood collection for a total of two surveys per day over 3 consecutive days. The symptoms on the survey are similar to that of the Rivermead instrument (31, 32) but with additional survey questions and responses relevant to operational blast OP training in addition to those of concussion (33). Participants were instructed to use a 5-point Likert scale (0 = “not experienced at all,” 1 = “no more of a problem than before training,” 2 = “mild problem—present but don't really notice and doesn't concern me,” 3 = “moderate problem—I can continue what I am doing, but I notice the problem,” 4 = “severe problem—constantly present, feels like it could affect my performance”).

### Serum Collection and Preparation

Venous blood was collected directly into BD Vacutainer SST™ Serum Separation Tubes (Fisher Scientific, Waltham, MA) prior to (pre-OP: −3 to −2 h) and after (post-OP: +0.45 to +3 h) OP exposure to mirror DANA assessment. Serum was processed within 30 min according to the manufacturer's instructions. Samples were centrifuged at 1,000 × g for 10 min at room temperature. Samples were stored in 1-mL aliquots, supplemented with HALT protease/phosphatase inhibitors (Fisher Scientific, Waltham, MA) and stored at −80°C until use.

### Quantitative Biomarker Measurements

Glial fibrillary acidic protein (GFAP), ubiquitin carboxyl-terminal esterase L1 (UCH-L1), neurofilament light polypeptide (Nf-L), tau, and amyloid beta (Aβ)-40 and−42 were measured by multiplex digital immunoassay using a single molecule array technology with the SiMoA HD-1 instrument (Quanterix Corporation, Billerica, MA). All assays were performed based on manufacturer's recommendations as previously reported (34). Briefly, serum was thawed on ice, then centrifuged at 10,200 × g for 10 min at 4°C. Thereafter, 120 μL of serum supernatant was directly loaded onto a 96-well plate and diluted 1/4 during the assay. Each serum sample, standard, or internal control was tested in duplicate. All reported biomarker values were within the limits of detection reported, and internal quality controls were consistent (CV <1%) for each biomarker tested (Supplementary Table 2A). Inter- or intra-participant variation is provided for the biomarker analysis (Supplementary Table 2B). Curve fitting analysis was conducted using preset programs designed by the manufacturer.

### Data Management and Statistical Analysis

Data analysis was conducted with Prism version 8.2.1 (GraphPad, La Jolla, CA) under the assumptions that all participants underwent similar OP exposure conditions although barrel length and bullet weights are known to affect the exposure conditions for weapon systems. DANA metrics (SRT, PRT, and GNG) and biomarker levels are displayed as the median + interquartile range (IQR). Quantitative measurements were tested for normality and found to fit a non-normal distribution per the D'Agostino & Pearson normality test. Data was evaluated using non-parametric RM-ANOVA (Friedman's Test) with Dunn's *post-hoc* test for multiple comparison across days and within-subjects per day (levels: pre-D1, post-D1, pre-D2, post-D2, pre-D3, post-D3) (biomarker concentration [pg/mL], ^*^p ≤ 0.05). For comparisons between OP levels (peak psi or cumulative impulse) or symptoms, biomarker values were transformed into a delta (post- minus pre-OP exposure) prior to determining the two-tailed Spearman rank correlation coefficient (^*^p ≤ 0.05).

## Results

Demographic information of participants, the time of serum sample collection and survey assessment as well as the peak pressure or impulse values derived from left shoulder B3 sensors are shown (Table 1). Participants (*n* = 15) were males aged 33–52 (mean +/– SD: 42.3 +/– 5.7 years) with variable duration of service (mean +/– SD: 14.7 +/– 6.7 years; range: 8–26 years). DANA, symptom reporting, and serum sampling occurred before (pre-OP: mean: −2.48 h, range: −3.16–−2.10) and after (post-OP: mean: 1.40 h, range: 0.46–2.90 h) .50-caliber rifle firing. Peak pressures for D1 (mean +/– SD: 3.86 +/– 1.04 psi) and D2 (mean +/– SD: 3.82 +/– 0.42 psi) were similar but slightly elevated on D3 (mean +/– SD: 4.52 +/– 1.59 psi). Cumulative impulse values were highest on D1 (mean +/– SD: 42.2 +/– 18.7 psi-ms), compared to D2 (mean +/– SD: 19.3 +/– 7.37 psi-ms) and D3 (mean +/– SD: 34.1 +/– 13.4 psi-ms).

**Table 1:** Demographic data of military personnel exposed to overpressure from munitions.

**Number of subjects (*n*)**	15		
**Age (years)**			
Mean (SD)	42.3 (5.7)		
Range [Min–Max]	33–52		
**Duration of Service (years)**			
Mean (SD)	14.7 (6.7)		
Range [Min–Max]	8–26		
**Sample Collection Time (h)**			
**Event**	**Pre-exposure**	**Post-exposure**	
Mean (SD)	−2.48 (0.59)	1.40 (1.3)	
Range [Min–Max]	−3.16 to −2.1	0.46 to 2.90	
**Peak pressure (psi)**	**Day-1**	**Day-2**	**Day-3**
Mean (SD)	3.86 (1.04)	3.82 (0.42)	4.52 (1.59)
**Cumulative Impulse** **(psi** **×** **time) (milliseconds)**	**Day-1**	**Day-2**	**Day-3**
Mean (SD)	42.2 (18.7)	19.3 (7.37)	34.1 (13.4)

*Demographic Characteristics of Study Participants and Biosample Collection Timelines. Participants' age, duration of service, as well as pre-OP and post-OP exposure time-point for DANA, blood collection, and symptom surveys are indicated. Levels of OP exposure derived from left shoulder B3 sensors from all participants are displayed as the peak pressure (psi) and cumulative impulse (psi × time) levels are displayed for each day (mean + SD each day, based on all participants)*.

**Figure 1:**
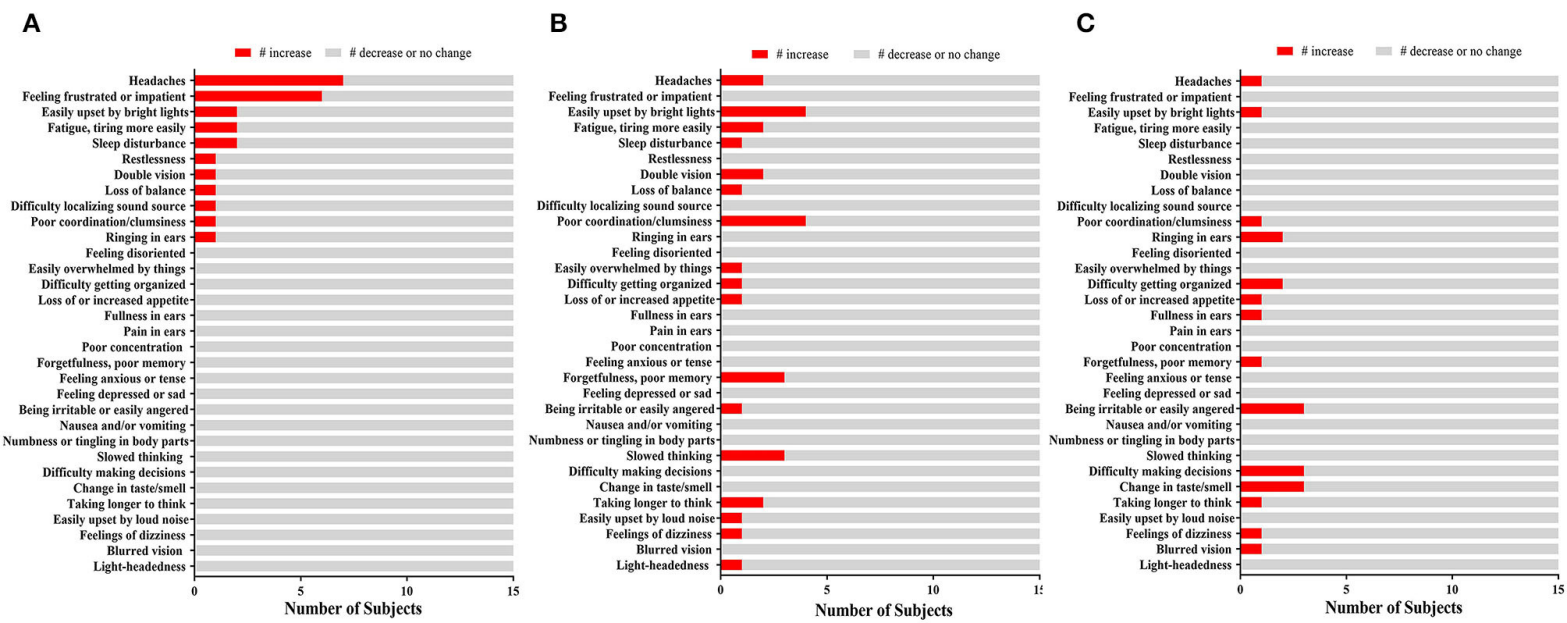
The Effect of OP Exposure on Symptom Reporting. Bar chart indicating the number of participants (*x*-axis) and self-reported symptoms (*y*-axis) on **(A)** Day 1, **(B)** Day 2, **(C)**, and Day 3. Those who reported an increase (red) compared those who did not (gray) are shown.

Neurocognitive assessment was performed using the DANA. SRT, PRT, and GNG assessments were generally not negatively (or adversely) affected by OP exposure (Supplementary Table 3). Symptom reporting was highly variable over time (Figure 1). Headaches (7/15, 47%) and feeling frustrated or impatient (6/15, 40%) were the two most frequently reported after OP events on D1. On D2 and D3, the above symptoms resolved. Few subjects reported other symptoms as a consequence of OP events during training. Therefore, symptoms were not further evaluated as a consequence of peak pressure, impulse levels, or for relationships with changes in biomarker levels.

**Figure 2:**
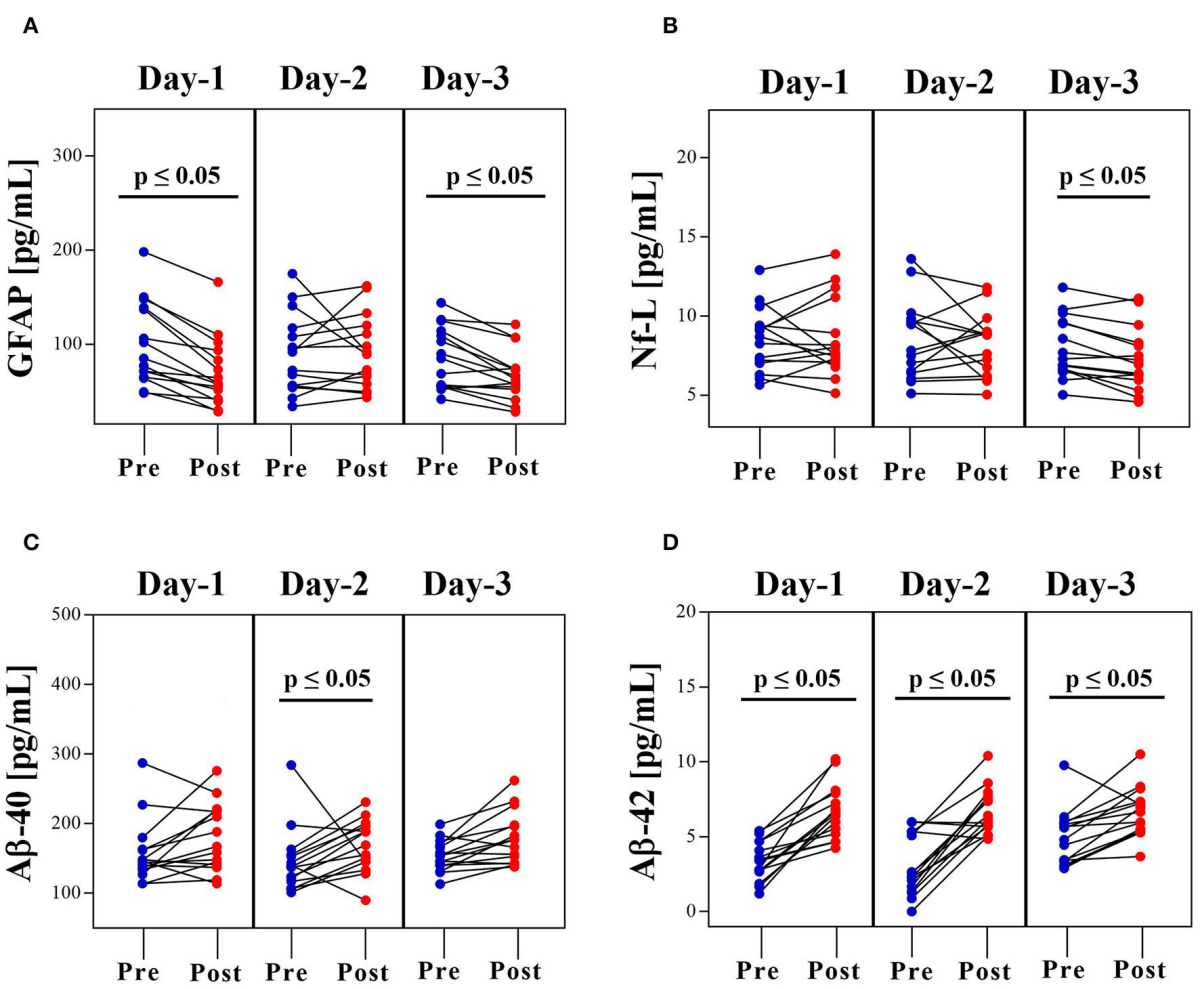
Acute Dynamics of Serum Protein Biomarkers before and after OP Exposure. Concentration of **(A)** GFAP, **(B)** Nf-L, **(C)** Aβ-40, and **(D)** Aβ-42 in serum collected before (pre-OP range: −3.16 to −2.10) and after (post-OP range: 0.45–3 h) OP exposure. Data is shown as concentration [pg/mL] (RM-ANOVA with Dunn's *post-hoc* test).

Next, TBI-related biomarkers were measured in serum collected before and after daily OP (Figure 2). The Dunn's multiple comparison test for daily changes in biomarkers are shown (Supplementary Table 4). GFAP levels fell significantly on D1 (pre-OP—median: 84.7, IQR: 64.8–139 pg/mL; post-OP—median: 58.3, IQR: 41.8–93.4 pg/mL, *p* ≤ 0.05). Values were not affected on D2 (pre-OP—median: 91.8, IQR: 55.9–117 pg/mL; post-OP—median: 89.2, IQR: 57.6–120 pg/mL, *p* = NS) but were suppressed again on D3 (pre-OP—median: 84.8, IQR: 55.2–114 pg/mL; post-OP—median: 62.4, IQR: 51.9–74.3 pg/mL, *p* ≤ 0.05) (Figure 2A). Nf-L levels were not affected (D1: pre-OP—median: 8.69, IQR: 7.03–9.43 pg/mL; post-OP—median: 7.69, IQR: 7.07–11.2 pg/mL, NS; D2: pre-OP—median: 7.82, IQR: 6.4–9.84 pg/mL; post-OP—median: 8.8, IQR: 6.21–9.02 pg/mL, NS) until D3 of OP exposure (pre-OP—median: 7.29, IQR: 6.63–9.60 pg/mL; post-OP—median: 6.94, IQR: 5.95–8.3 pg/mL, *p* ≤ 0.05) (Figure 2B).

OP-mediated elevation of Aβ peptide levels was more robust. Aβ-40 showed a somewhat progressive, upward trend each day (D1: pre-OP—median: 146, IQR: 134–163 pg/mL; post-OP—median: 167, IQR: 141–219 pg/mL, NS; D2: pre-OP—median: 138, IQR: 117–155 pg/mL; post-OP—median: 169, IQR: 145–196 pg/mL, *p* ≤ 0.05; D3: pre-OP—median: 154, IQR: 140–169 pg/mL; post-OP—median: 177, IQR: 143–198 pg/mL, NS) (Figure 2C). Similarly, Aβ-42 levels were also higher as a consequence of daily OP exposure (D1: pre-OP—median: 3.41, IQR: 2.67–4.7 pg/mL; post-OP—median: 6.52, IQR: 5.19–7.89 pg/mL, *p* ≤ 0.05; D2: pre-OP—median: 2.31, IQR: 1.34–5.32 pg/mL; post-OP—median: 6.17, IQR: 5.68–7.71 pg/mL, *p* ≤ 0.05; D3: pre-OP—median: 4.81, IQR: 3.35–5.93 pg/mL; post-OP—median: 6.66, IQR: 5.34–7.36 pg/mL, *p* ≤ 0.05) (Figure 2D). Further, Aβ-42/Aβ-40 ratios showed a significant upward trend after the each consecutive OP exposure event (D1: pre-OP—median: 0.021, IQR: 0.016–0.029 pg/mL; post-OP—median: 0.037, IQR: 0.031–0.043 pg/mL, *p* ≤ 0.05; D2: pre-OP—median: 0.018, IQR: 0.012–0.030 pg/mL; post-OP—median: 0.038, IQR: 0.033–0.045 pg/mL, *p* ≤ 0.05; D3: pre-OP—median: 0.030, IQR: 0.023–0.037 pg/mL; post-OP—median: 0.037, IQR: 0.031–0.042 pg/mL, *p* ≤ 0.05) (Supplementary Figure 1A). There were no appreciable changes in the levels of UCH-L1 (range: 0–30.9 pg/mL) (Supplementary Figure 1B) or tau (range: 0–0.502 pg/mL) (Supplementary Figure 1C) on either day.

The changes in biomarker levels were compared to peak OP and cumulative impulse levels on each training day. Spearman rank correlations are indicated for all comparisons (Table 2). On D1, peak pressure (psi) levels were directly proportional to increased dNf-L (*r* = +0.55, *p* = 0.03) although the median response in levels detected pre-OP and post-OP were not significant as previously shown. Biomarker changes were not associated with peak-OP or cumulative impulse values evaluated on D2. In contrast, the negative change in dGFAP was aligned with peak OP (*r* = −0.54, *p* = 0.04) and dAβ-42 (*r* = +0.65, *p* = 0.01) correlated to cumulative impulse levels on D3. The highest cumulative impulse levels from rifle fire on D1 were associated with increased serum Nf-L (positive dNf-L). On D2, the lowest cumulative impulse levels were recorded and did not correlate to changes in biomarker levels. In contrast, the second highest cumulative impulse levels recorded on D3 were in accordance with a drop in GFAP (e.g., negative dGFAP) and elevation of Aβ-42 (e.g., positive dAβ-42). Next, the cumulative effect of exposures (Sum-OP or Sum-impulse) was examined by comparing changes in biomarker levels on D3 post-OP to those measured in blood collected on D1 pre-OP to OP or impulse levels on D1–D3. Spearman rank correlations are indicated for all comparisons (Table 3). Biomarker changes were not associated with the Sum-OP. The changes in dGFAP and dNf-L did not correlate with the Sum-impulse levels. However, dAβ-40 showed moderate correlation (*r* = +0.53, *p* = 0.042) and dAβ-42 showed strong correlation (*r* = +1.00, *p* = < 0.0001) with Sum-impulse. The changes in the biomarker levels were not significantly correlated with number of shots fired on either day (Supplementary Table 5). Finally, the changes in biomarker levels were not significantly correlated with SRT, PRT, or GNG DANA metrics or to symptoms reported by the study participants (data not shown).

**Table 2:** The relationship between peak OP levels (psi) and cumulative Impulse (psi-ms) and biomarker changes is indicated.

**Biomarker Change**		**Peak OP (psi)**		**Cumulative Impulse (psi-ms)**	
	**Day**	**Spearman *r***	***p*-value**	**Spearman *r***	***p*-value**
dGFAP	D1	−0.39	NS	−0.08	NS
	D2	0.11	NS	−0.44	NS
	D3	**−0.54**	**0.04**	−0.09	NS
dNfL	D1	**0.55**	**0.03**	−0.46	NS
	D2	0.10	NS	−0.25	NS
	D3	0.28	NS	0.01	NS
dAβ-40	D1	0.20	NS	−0.10	NS
	D2	−0.30	NS	−0.45	NS
	D3	0.32	NS	.50	NS
dAβ-42	D1	0.24	NS	−0.28	NS
	D2	−0.31	NS	−0.47	NS
	D3	0.20	NS	**0.65**	**0.01**
dAβ-42/40 Ratio	D1	−0.33	NS	0.11	NS
	D2	−0.21	NS	0.45	NS
	D3	−0.01	NS	0.10	NS

*The correlation coefficient is shown in bold (2-tailed, Spearman r)*.

**Table 3:** The relationship between the summations of either OP levels (psi) or impulse (psi-ms) determined during training (D1, D2, and D3) and biomarker changes defined by the last day of training (D3 Post-OP) to the first day before training (Day1 Pre-OP) is indicated.

**Biomarker Change (D3 Post OP–D1 Pre OP)**	**Summation OP (psi)**		**Summation Impulse (psi-ms)**	
	**Spearman *r***	***p*-value**	**Spearman *r***	***p*-value**
dGFAP	−0.32	NS	−0.27	NS
dNfL	−0.01	NS	0.18	NS
dAβ-40	0.37	NS	**0.53**	**0.042**
dAβ-42	0.14	NS	**1.0**	**< 0.0001**

*The correlation coefficient is shown in bold (2-tailed, Spearman r)*.

## Discussion

Repeated OP exposure is associated with nervous system–related health effects. Yet objective measurements that may be used to define a trauma effect, particularly in the acute timeframe that is applicable to monitoring, remain difficult to define. Therefore, this study evaluated acute serum biomarker profiles, neurocognitive metrics, and reported symptomology among participants exposed to daily OP from .50-caliber rifle fire during a 3-day training period.

Quantitation of peak pressure and/or impulse caused by gunfire (not the number of shots fired) is the definitive metric that represents exposures derived from rapid-fire weapons per DoD requirements associated with health assessment as provided by Health and Human Services, Centers of Disease Control, and the National Institute for Occupational Health and Safety (https://www.cdc.gov/niosh/hhe/reports/pdfs/2013-0124-3208.pdf). Our current study shows that low levels of OP from .50-caliber rifle fire training did not have a substantive negative effect upon neurocognitive subtasks from the DANA. The null change in SRT is not surprising. SRT is similar among concussed vs. non-concussed athletes (35), and the participants in this study were exposed to subconcussive overpressure conditions. The decrease in PRT and GNG subtasks is indicative of a slightly faster response that has been previously reported to occur among nearly 67% of study participants (29) and may be reflective of a “practice effect” among persons without a clinically defined concussion (36). Essentially, the participants are likely paying more attention to the task itself, which is common. Few subjects reported headaches, the top-ranked symptom. The levels recorded for median peak OP among all participants was <4 psi, which is often cited as a “safe level” in regards to disruption of the ear drum among subject matter experts (37). Repeated exposure to moderate-to-high OP (≥5 psi) leads to persistent changes in symptomology and neurocognitive performance (38, 39), which we found not to occur in these participants. Thus, the observations shown herein are aligned with recent studies indicating that exposure to low levels of OP from .50-caliber rifles is common and does not largely affect DANA or symptoms although precautions to avoid higher levels of exposure are suggested due to peak pressure (psi) exposure guidelines.

The association between repeated OP and TBI is a well-accepted hypothesis (2, 40). Therefore, biomarkers are generally expected to increase as a consequence of OP exposure. UCH-L1 is well-known to be elevated within 24 h after severe, not subconcussive, TBI (41–43). Nf-L and tau are typically elevated during much later timeframes and associated with CT abnormalities (25, 44, 45). Serum tau may also be elevated acutely after exposure to moderate psi levels in rodent models (46). This study shows that serum UCH-L1 and tau were generally unaffected by repeated OP exposure after .50-caliber rifle fire. The lack of a UCH-L1 or tau response after low-level OP in this context is fitting for a subconcussive exposure that lacks to the need to employ the concussion protocol or imaging as well an exposure paradigm that does not lead to a moderate-to-severe head wound.

A small (albeit potentially non-significant) increase in GFAP or Nf-L was also expected after daily OP exposure. Serum GFAP typically increases after acutely after moderate-to-severe TBI or CT-positive closed head injury (47). Among boxers, mild head impact is associated with higher levels of serum Nf-L in serum collected >7-days (48). In contrast, Nf-L and (to a greater extent) GFAP were found to be decreased in subjects exposed to subconcussive levels of OP in this study. The biological relevance of serum GFAP (D1 and D3) and Nf-L (D3 only) suppression as well as the correlation between decreased GFAP and peak OP levels is not yet known and potentially confounding, when viewed in the context of longer timeframes or clinically diagnosed, moderate-to-severe TBI as a predetermined endpoint. However, it is interesting to note that Nf-L levels decreased 1–12 h within serum collected from hockey players post–game play (25). This time-dependent observation is often overlooked and may indicate that acute (e.g., hourly) temporal dynamics of Nf-L may be useful in monitoring early phase OP-mediated effects when compared to a participant's basal biomarker levels, whereas leakage of a fairly large protein from the CNS is useful in the context of a known variable (e.g., clinical TBI diagnosis) during longer timeframes (e.g., weeks–months). Our group has previously reported that serum GFAP levels also decreased in accordance with persistent concussion-like symptomology after mild-to-moderate OP exposure caused by blast (34). Replication of both observations with larger cohorts and variation of peak OP levels and assessment of medical relevance compared to basal levels is ongoing.

Elevation of Aβ peptides was most robust after OP overpressure exposure due to .50-caliber rifle fire. These trends were generally associated with higher peak OP psi or impulse levels on the last day of training. In addition, Aβ-40 elevation correlated positively with the sum of impulse levels measured throughout the 3-day course, indicating that there may be a cumulative effect. This work is the first to explicitly report elevation of both Aβ-40 and−42 peptides within h of low-level OP exposure caused by rifle fire. Aβ-40 and−42 are well-understood as a key pathological component in chronic, neurodegenerative disease progression (49). Aβ levels are increased acutely among brain trauma patients, even those with diffuse injury compared to controls (50). Furthermore, Aβ-42 is higher in plasma exosomes collected from military personnel with mTBI compared to non-TBI controls (51). Transient leakage of small peptides, such as Aβ, from the CNS is feasible in the context of acute, subconcussive OP. Gap junctions and the blood–brain barrier are transiently affected by OP caused by blast in rodent models (52, 53). Therefore, acute elevation of amyloid beta peptides in serum may be sensitive responders of low-level, subconcussive OP. A potential caveat is that Aβ peptides and their precursor, APP, are also expressed outside of the CNS, including the epidermis, adipose tissue, and muscle (54, 55) and may be affected by circadian rhythms (56), leading to changes throughout the day (57). Tissue injuries were not reported and sleep disturbance were not significant among participants. Thus, circadian rhythms are not a likely confound. Overall, the changes in serum Aβ levels may have utility as biomarkers of low-level OP caused by .50-caliber rifle fire.

This preliminary work is not without a few limitations that are common to sampling within the context of real-world scenarios. First, the overall sample size (*N* = 15) is small. The participants of this study consist of an extraordinarily unique group due to the nature and context of specialized operational training. Second, the biomarker values are variable among non-injured controls or presumably healthy individuals (58) due to expected biological variance, how or which samples are defined as controls (59) and which quantitative assays (e.g., colorimetric vs. digital) are used (60, 61). Third, Aβ-40 and−42 peptides are produced by a wide variety of tissues inclusive of the CNS and periphery (62). In addition, Aβ peptide fluctuation may occur due to diet, medication, and stress. The current study cannot distinguish between the central and peripheral sources of the biomarker changes. Additional studies are in progress to address these potential confounds. To that end, we found that biomarker levels detected in commercially available controls were lower compared to levels among participants of this study and a previous report (38). Incorporation of control groups consisting of participants who conduct the same activities without firing .50-caliber weapons would be highly valuable. Due to the requirement that each participant actively engages in training, these groups do not yet exist. Instead, this study is structured such that each participant is compared to himself before OP exposure. A means to establish control groups that consist of personnel similar to those within this study that meets study guidelines, training requirements, and availability of participants is in progress. Lastly, future studies will seek to evaluate of biomarker changes in the context of operationally safe (<4 psi) or above safe (≥4 psi) peak pressure (63) as well as low (<25 psi-ms) vs. high (≥25 psi-ms) impulse levels, which has been suggested by a working group, but impulse thresholds are experimental (39).

## Conclusions

Subconcussive, low-level OP exposure caused by .50-caliber rifle fire is associated with daily fluctuation of serum biomarkers although symptomology is infrequent. This preliminary work indicates that acute elevation of Aβ peptides (and the Aβ-42/40 ratio) in serum may have utility as biomarkers of subconcussive OP relevant to impulse levels and that measurement of basal or pre-OP exposure biomarker levels serve as a reference for acute, post-OP exposure effects. The rifle systems and ammunition used among study participants offer authentic, real-world, scenarios akin to those that are likely to occur in combat. Therefore, assessment of dynamic biomarker changes, particularly when evaluated before and after OP exposure in the absence of a clinically defined TBI or concussion, are poised for additional evaluation and may be adaptable to operationally relevant health monitoring.

## Data Availability Statement

All datasets generated for this study are included in the article/Supplementary Material.

## Ethics Statement

The protocol was approved by the Walter Reed Army Institute of Research Institutional Review Board (WRAIR protocol #2304). The participants provided their written informed consent to participate in this study.

## Author Contributions

AB, CL, ME, and GK designed the study. CL, ME, and GK conducted DANA, survey, biological sample collection, and data collection. BT conducted biological sample processing and SiMoA biomarker assays. BT, AB, CL, and JN conducted data management and statistical analysis. BT, AB, CL, ME, and GK prepared the manuscript. All authors contributed to the article and approved the submitted version.

## Conflict of Interest

The authors declare that the research was conducted in the absence of any commercial or financial relationships that could be construed as a potential conflict of interest.

## Supplementary Material

Supplementary Figure 1 Acute Dynamics of Additional Serum Protein Biomarkers before and after OP Exposure. Graphs are shown for the **(A)** the Aβ 42/40 ratio as well as concentrations of **(B)** UCH-L1 and **(C)** Tau in serum collected before (pre-OP range: −3.16 to −2.10) and after (post-OP range: 0.45–3 h) OP exposure. UCH-L1 and tau data is shown as the concentration [pg/mL] (^*^*p* ≤ 0.05, RM-ANOVA with Dunn's *post-hoc* test).

Supplementary Table 1 Detailed Characteristics of Study Participants and Study Outcome Metrics.Each participants' age, duration of service, peak overpressure (psi), and cumulative impulse from left shoulder B3 sensors (time and delta), pre-OP and post-OP exposure time-points (time and delta), DANA metrics (milliseconds and delta), individual serum biomarker levels (pg/mL and delta), and transformed symptom survey responses (0 = decrease or no change; +1 = increase) are shown.

Supplementary Table 2 Variation in Biomarker Analysis. **(A)** Assay limits are shown as provided by the manufacturer. **(B)** The coefficient of variation (CV %) in the biomarker assays is shown for internal quality controls (QC1 and QC2), the expected concentration is provided by Quanterix per assay lot. **(C)** Daily coefficient of variation of biomarker concentrations among study participants.

Supplementary Table 3 The Effect of OP Exposure on DANA Metrics. Values (milliseconds, ms) of (A) SRT (B) PRT (C) GNG were determined before (pre-) and after (post-) OP exposure for each participant and each day is displayed as the median + interquartile range (IQR) (^*^*p* ≤ 0.05, RM-ANOVA with Dunn's *post-hoc* test).

Supplementary Table 4 Dunn's multiple comparisons for biomarkers across three days (^*^*p* ≤ 0.05, RM-ANOVA with Dunn's *post-hoc* test).

Supplementary Table 5 The relationship between number of shots fired each day and biomarker changes is indicated. (^*^*p* ≤ 0.05, 2-tailed, Spearman *r*).
