# Supplementary figures and images for: Overpressure Exposure From .50-Caliber Rifle Training Is Associated With Increased Amyloid Beta Peptides in Serum
Source: Front Neurol. 2020 Jul 24;11:620. doi: 10.3389/fneur.2020.00620 (PMC7396645; doi:10.3389/fneur.2020.00620)

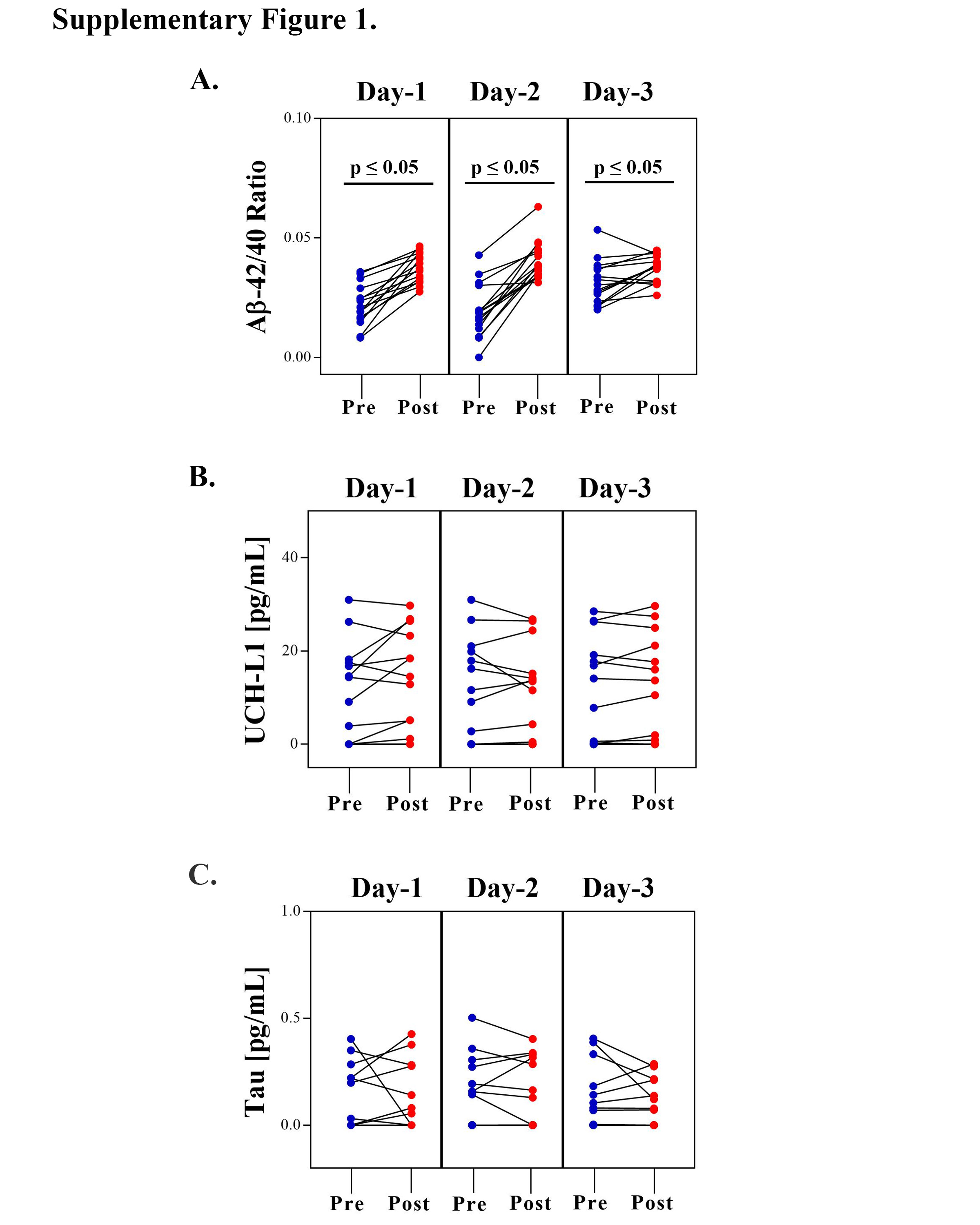

Supplement: Supplementary Figure 1 — Acute Dynamics of Additional Serum Protein Biomarkers before and after OP Exposure. Graphs are shown for the (A) the Aβ 42/40 ratio as well as concentrations of (B) UCH-L1 and (C) Tau in serum collected before (pre-OP range: −3.16 to −2.10) and after (post-OP range: 0.45–3 h) OP exposure. UCH-L1 and tau data is shown as the concentration [pg/mL] (*p ≤ 0.05, RM-ANOVA with Dunn's post-hoc test). [file Image_1.JPEG]
