# Supplementary material for: Overpressure Exposure From .50-Caliber Rifle Training Is Associated With Increased Amyloid Beta Peptides in Serum
Source: Front Neurol. 2020 Jul 24;11:620. doi: 10.3389/fneur.2020.00620 (PMC7396645; doi:10.3389/fneur.2020.00620)
Supplement: Supplementary Table 3 — The Effect of OP Exposure on DANA Metrics. Values (milliseconds, ms) of (A) SRT (B) PRT (C) GNG were determined before (pre-) and after (post-) OP exposure for each participant and each day is displayed as the median + interquartile range (IQR) (*p ≤ 0.05, RM-ANOVA with Dunn's post-hoc test). [file Table_3.DOCX]

| **Supplementary Table 3.** | | | | | | |
| --- | --- | --- | --- | --- | --- | --- |
| **DANA (milliseconds)** | **Pre-OP** | | **Post-OP** | |  | |
|  | **Median** | **IQR** | **Median** | **IQR** | **% Change** | **p-value** |
| **SRT** |  | | | | | |
| Day-1 | 274.6 | 238.2-308.3 | 260.4 | 245.8-314.7 | -5.17 | NS |
| Day-2 | 284.6 | 261.3-341.4 | 303.6 | 242.9-351.5 | 6.68 | NS |
| Day-3 | 287.7 | 264.3-328.4 | 274.6 | 265.3-306 | -4.55 | NS |
| **PRT** |  | | | | | |
| Day-1 | 656.8 | 546.6-684.3 | 601.8 | 525.6-664.5 | -8.37 | NS |
| Day-2 | 663.3 | 613-710.4 | 660.7 | 646.3-686.4 | -0.39 | NS |
| Day-3 | 617.8 | 552.8-667.6 | 609.5 | 578.5-632.5 | -1.34 | NS |
| **GNG** |  | | | | | |
| Day-1 | 587.8 | 568.5-655.4 | 574.1 | 537.5-589.6 | -2.33 | NS |
| Day-2 | 604.4 | 558.6-636.9 | 585.6 | 545.1-630.1 | -3.11 | NS |
| Day-3 | 594.8 | 576.1-623.7 | 616.5 | 548.8-642.1 | 3.65 | NS |

**Table Legend**

**Supplementary Table 3.** The Effect of OP Exposure on DANA Metrics. Values (milliseconds, ms) of (A) SRT (B) PRT (C) GNG were determined before (pre-) and after (post-) OP exposure for each participant and each day is displayed as the median + interquartile range (IQR) (* p ≤ 0.05, RM-ANOVA with Dunn’s post-hoc test).
