# Supplementary material for: Overpressure Exposure From .50-Caliber Rifle Training Is Associated With Increased Amyloid Beta Peptides in Serum
Source: Front Neurol. 2020 Jul 24;11:620. doi: 10.3389/fneur.2020.00620 (PMC7396645; doi:10.3389/fneur.2020.00620)
Supplement: Supplementary Table 5 — The relationship between number of shots fired each day and biomarker changes is indicated. (*p ≤ 0.05, 2-tailed, Spearman r). [file Table_5.DOCX]

**Supplementary Table 5.**

|  |  | **Shots fired** | |
| --- | --- | --- | --- |
| **Biomarker Change** | **Day** | **Spearman *r*** | **p-value** |
| **dGFAP** | D1 | -0.06 | NS |
|  | D2 | -0.14 | NS |
|  | D3 | -0.04 | NS |
| **dNfL** | D1 | -0.20 | NS |
|  | D2 | -0.08 | NS |
|  | D3 | -0.12 | NS |
| **dAβ-40** | D1 | -0.06 | NS |
|  | D2 | -0.03 | NS |
|  | D3 | 0.20 | NS |
| **dAβ-42** | D1 | -0.51 | NS |
|  | D2 | -0.10 | NS |
|  | D3 | 0.32 | NS |

**Table Legend**

**Supplementary Table 5**. The relationship between number of shots fired each day and biomarker changes is indicated. (* p ≤ 0.05, 2-tailed, Spearman r).
